# Patient Characteristics and Clinical Process Predictors of Patients Leaving Without Being Seen from the Emergency Department

**DOI:** 10.5811/westjem.2020.6.47084

**Published:** 2020-08-25

**Authors:** Niels K. Rathlev, Paul Visintainer, Joseph Schmidt, Joeli Hettler, Vanna Albert, Haiping Li

**Affiliations:** * University of Massachusetts Medical School – Baystate, Department of Emergency Medicine, Springfield, Massachusetts; † University of Massachusetts Medical School – Baystate, Department of Epidemiology and Biostatistics Core, Springfield, Massachusetts

## Abstract

**Introduction:**

Delays in patient flow in the emergency department (ED) result in patients leaving without being seen (LWBS). This compromises patient experience and quality of care. Our primary goal was to develop a predictive model by evaluating associations between patients LWBS and ED process measures and patient characteristics.

**Methods:**

This was a cross-sectional study in a 95,000 annual visit adult ED comparing patients LWBS, with controls. Data were drawn from four seasonally adjusted four-week periods (30,679 total visits). Process measures included 1) arrivals per hour; 2) “door-to-provider” time; and the numbers of 3) patients in the waiting room; 4) boarding ED patients waiting for an inpatient bed; 5) providers and nurses (RN); and 6) patients per RN. Patient characteristics collected included 1) age; 2) gender; 3) race/ethnicity; 4) arrival mode (walk-in or via emergency medical services [EMS]); and 5) acuity based on Emergency Severity Index (ESI). Univariable analyses included t-tests and Pearson’s chi-square tests. We split the data randomly into derivation and validation cohorts. We used backward selection to develop the final derivation model, and factors with a p-value ≤ 0.05 were retained. Estimates were applied to the validation cohort and measures of discrimination (receiver operating characteristic) and model fit were assessed.

**Results:**

In the final model, the odds of LWBS increased with the number of patients in the waiting room (odds ratio [OR] 1.05; 95% confidence interval [CI], 1.03 to 1.06); number of boarding patients (OR 1.02; 95% CI, 1.01 to 1.03); arrival rate (OR 1.04; 95% CI, 1.02 to 1.05) and longer “door-to-provider” times (test of linear trend in the adjusted OR was p = 0.002). Patient characteristics associated with LWBS included younger age (OR 0.98; 95% CI, 0.98 to 0.99), and lower acuity (higher ESI category) (OR 2.01; 95% CI, 1.84 to 2.20). Arrival by EMS was inversely associated with LWBS (OR 0.29; 0.23 to 0.36). The area under the curve for the final model in the validation cohort was 0.85 (95% CI, 0.84 to 0.86). There was good agreement between the observed and predicted risk.

**Conclusion:**

Arrival rate, “door-to-provider time,” and the numbers of patients in the waiting room and ED boarders are all associated with patients LWBS.

## INTRODUCTION

Delays in care in the emergency department (ED) lead to a higher number of patients who leave without being seen (LWBS), ie, who are triaged and register for care but subsequently leave without any evaluation by a provider.^1^ On occasion, these patients suffer from significant illnesses or injuries and would, in hindsight, have benefited from time-sensitive medical interventions including emergency care. This compromises not only patient experience, but also safety, quality of care, and risk management. Many of the patients who LWBS do so because of delays in being seen, and up to 70% seek attention within 24 hours of leaving, either by returning to the ED or by presenting to alternative sites of medical care.^2^–^3^ Finally, high rates of LWBS negatively impact institutional revenue and may present a significant financial loss to the institution.^4^–^5^

High LWBS rates are predictably a challenge in large teaching institutions in metropolitan areas. High acuity and volume are associated with increased ED length of stay and rates of ED boarding and “left before completing treatment”; 6–7 non-profit institutions often compare unfavorably with for-profit competitors on these measures.^8^–^9^ Consequently, it is important to appreciate that hospitals have different baselines of performance that may be tied to volume and capacity, rather than quality of care. Notably, the current study was performed in a Level I trauma center teaching institution, which is the only tertiary-care referral center in a large, four-county area in western Massachusetts. It is one of the busiest EDs in New England based on annual volumes and has exceptionally high acuity based on the 2018 Association of Academic Chairs of Emergency Medicine Annual Survey. Our ED ranked above the 75%ile in both annual volume and rate of LWBS compared with national medians on this survey.

The associations between LWBS rates, crowding, boarding of admitted patients in the ED, and delays in care have been described but continue to resist solution.^10^ Our aim was to evaluate associations between individuals who LWBS and ED process measures and patient characteristics, and to derive and validate a predictive model. We undertook this endeavor by performing a cross-sectional comparative study with the goal of developing a highly predictive model for discriminating patients who LWBS from those who initiate evaluation and treatment by a provider.

## METHODS

### Setting

The study was performed at *x* Medical Center in *y, z* in the medical-surgical (non-psychiatric) adult ED, which is comprised of 66 licensed bays. Pediatric patients were excluded from the study. In 2015, 95,000 annual visits were seen in the adult ED with a baseline LWBS rate of 7.0%. The “provider in triage” model was not implemented during the study period. We defined a patient as LWBS if the individual received, at minimum, an abbreviated triage consisting of 1) reason for ED visit and age; 2) registered for care but subsequently left without full registration or evaluation, ie, history or physical exam, by an advanced practitioner (AP) or physician. The patient was documented as LWBS after being called in the waiting room or treatment area with no response to overhead announcement on three separate occasions at 15-minute intervals. We typically did not know the exact time of LWBS unless the patient specifically informed the staff of his or her intent, but this was the exception and not the rule. Data was collected from our electronic health record and tracking system Cerner (North Kansas City, MO) on an hourly basis since process measures can vary significantly over longer time periods. We documented the relevant data at the beginning of every hourly interval starting with 00:00. Our goal was to evaluate the associations between individuals LWBS and 1) patient characteristics, and 2) process measures related to throughput and staffing.

**Population Health Research Capsule**

What do we already know about this issue?

Specific patient characteristics, new patient arrivals, and boarding hours are associated with patients leaving without being seen (LWBS).

What was the research question?

Can we derive and validate a highly predictive model of a patient LWBS?

What was the major finding of the study?

We validated a model with “very good” discrimination that included both patient characteristics and clinical process indicators.

How does this improve population health?

The model can be used in real time to predict whether a patient presenting for emergency care is likely to LWBS.

### Study Design

We employed a cross-sectional study design in which patients who LWBS were compared with controls who initiated evaluation and treatment by an AP or physician. To control for seasonal variability, data were drawn from four four-week periods (30,679 total visits) in September–December 2015 and March–June 2016. Patients were eligible for inclusion if they registered in the ED at any time during the indicated periods. Patients were excluded from analysis if they arrived under police escort or died in the ED.

### Measured Variables

Patient characteristics collected included age, gender, race/ethnicity (White, Hispanic, Black, Asian, or other/not indicated), arrival mode (walk-in or via emergency medical services [EMS]), acuity based on Emergency Severity Index (ESI) level, month of presentation (ie, March, June, September or January) and time of registration (categorized into four six-hour time periods). Insurance status was not included as a variable because patients had not completed the registration process, and their insurance status was not documented at the time they LWBS.

Process measures of ED utilization and resource allocation were routinely collected every hour.^11^ These measures included the numbers of 1) patients in the waiting room; 2) patients in treatment bays (licensed ED bays plus hallway beds); and 3) boarders, ie, admitted patients waiting for an in-patient bed. The staff members on duty included the following: 1) attending physicians; 2) advanced practioners (AP); 3) emergency medicine residents; 4) registered nurses (RN) – our ED does not employ licensed practical nurses; and 8) patient care technicians who perform vital signs, obtain laboratory samples including blood draws and electrocardiograms, etc. The ratio of the numbers of patients per RN was computed based on patients in ED treatment bays only. The arrival rate of patients was measured for the 60-minute period in which study subjects presented. Finally, we measured the “door-to-provider time” (attending physician, resident, or AP) in 30-minute increments starting with the initial ED presentation. We chose 30-minute increments because previous literature has concluded that delays of 30 or 60 minutes appear to be critical time periods for patients when deciding to LWBS.^12^

### Statistical Analysis

Preliminary descriptive analyses included means and standard deviations, medians and ranges for continuous variables. We described categorical variables using frequency distributions. Univariable approaches included t-tests for continuous data and Pearson’s chi-square for categorical data. Since our sample size was large, we split the data into derivation (n = 14,937) and validation cohorts (n = 14,445) in order to assess the fit of the model. The data were split randomly and were approximately balanced on the number of days from each month.

We used generalized estimating equations (GEE) with a binomial family and logit link to derive parameter estimates. Further, the GEE model clustered on day of registration (to account for day of the week) and employed robust standard errors. We used backward selection to develop a final model based on the derivation cohort (n = 14,937). Beginning with a model that included all variables, the least significant of those remaining was removed in an iterative fashion. Any process measures – related to utilization and resource allocation – with a p-value ≤ 0.05 were retained in the final model. The same was true of patient characteristics that met this criterion. The model that emerged from the backward selection process was compared to other model configurations of utilization variables that were considered to potentially capture LWBS risk. The final model was selected from among these comparisons using significance testing of variables for nested models or by way of a modified Akaike information criterion (AIC) for GEE models, as recommended by Pan and implemented in Stata by Cui.^13^–^14^

We then evaluated the final model from the derivation cohort in the validation cohort (n = 14,445). Discrimination was evaluated using a receiver operating characteristic (ROC) curve with 95% confidence intervals. Calibration was represented using a plot of the observed vs predicted risk of LWBS over deciles of categories. We also assessed calibration fit by computing the integrated calibration index (ICI).^15^ The ICI computes the difference between the observed and predicted probabilities over the range of predicted probabilities. Estimates of the mean, median, and the maximum absolute difference, *E_max_*, are provided.^16^ Statistical analyses were conducted in Stata v15.1 (StataCorp, College Station, TX) and R (https://www.R-project.org/; Foundation for Statistical Computing, Vienna, Austria). The study was approved by the institutional review board of Baystate Medical Center.

## RESULTS

A total of 30,679 patients visited the ED during the four-month study period. We calculated the following mean data for the study population: 1) 82 admissions per day; 2) 251 patients presenting per day; 3) 7.2% LWBS rate; and 4) 2.9% ESI 1; 40.8% ESI 2; 38.2% ESI 3; 17% ESI 4; 1.1% ESI 5. After removing 1297 observations due to exclusion criteria and missing data, 29,382 patients (95.8%) were available for study. In this cohort of 29,382 individuals, a total of 2,213 patients (7.5%) LWBS. Tables 1 and 2 show the description of the derivation cohort (n = 14,937) and differences between patients who did and did not LWBS. A total of 1122 (7.5%) patients LWBS. Although p-values for the comparisons of the two groups were statistically significant, absolute differences between the groups were generally small. There was a significant increase in the proportion of patients LWBS as the “door-to-provider” time increased in 30-minute increments.

Table 3 shows the adjusted odds ratios (OR) for the variables that were retained in the final regression model based on the derivation cohort. Patient characteristics associated with LWBS included younger age (OR 0.98; 95% CI, 0.98 to 0.99) and lower acuity (higher ESI category) (OR 2.01; 95% CI, 1.84 to 2.20). Arrival by EMS was inversely associated with LWBS (OR 0.29; 0.23 to 0.36). In general, the odds of LBWS increased as clinical demand increased, as measured by number of patients in the waiting room (OR 1.05; 95% CI, 1.03 to 1.06), number of patients in treatment bays (OR 1.02, 95% CI, 1.01 to 1.02), number of boarding patients (OR 1.02; 95% CI, 1.01 to 1.03), and arrival rate (OR 1.03; 95% CI, 1.02 to 1.05). Adjusting for all other factors in the model, the odds of LWBS increased with longer “door-to-provider” times (measured in 30-minute increments). For this measure, a test of a linear trend in the adjusted ORs was significant at p < 0.002.

**Figure 1:**
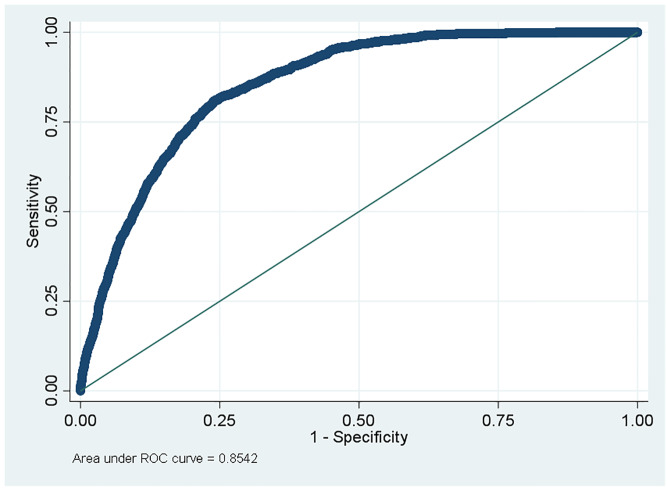
Validation cohort – receiver operating characteristic curve. Area under the curve = 0.854. *ROC*, receiver operating characteristic; *AUC*, area under curve.

**Figure 2:**
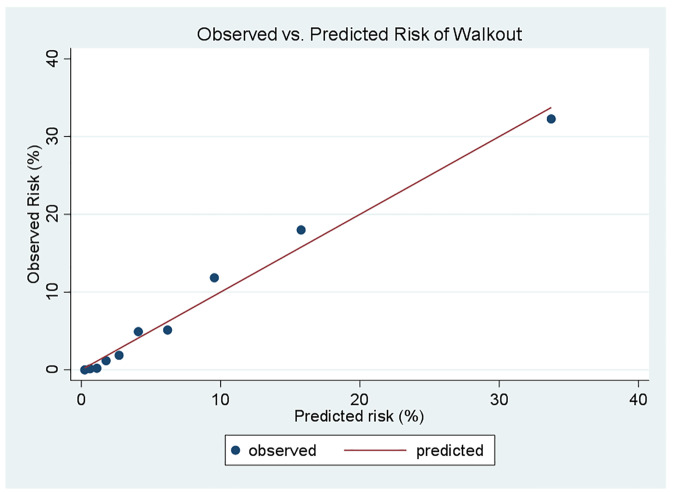
Calibration plot of observed vs predicted risk of patients leaving without being seen.

Parameter estimates from the derivation cohort were applied to the validation cohort and measures of discrimination and model fit were assessed. Figure 1 shows the receiver operating characteristic (ROC) curve for discriminating patients who LWBS from those for whom evaluation and treatment by a provider was initiated. The model has “very good” discrimination as indicated by an area under the curve (AUC) of 0.85 (95% CI, 0.84 to 0.86). Figure 2 presents a plot of the observed vs predicted risk over deciles of the predicted risk. The plot shows good agreement between the observed risk and what was predicted by the model. To further assess model calibration, we also computed the integrated calculation index (ICI) (mean absolute difference) and associated measures.^1^–^2^ The ICI and median (E_50_) absolute difference between the observed and predicted probabilities over the range of predicted probabilities were 0.009 and .005, respectively. These estimates indicate that on average model predictions are nearly identical to observed probabilities. The 90^th^ percentile (E_90_) and maximum difference (E_max_) were 0.03 and 0.12, respectively. Thus, 90% of the differences between the observed and predicted probabilities were no larger than about three absolute percentage points. The largest absolute difference between observed and expected probabilities was 12%. The mean (ICI) and median (E_50_) absolute difference between the observed and predicted probabilities were 0.009 and .005, respectively. The 90^th^ percentile (E_90_) and maximum difference (E_max_) were 0.03 and 0.12, respectively. The largest absolute difference between observed and expected probabilities was 12%.

## DISCUSSION

The unique contribution of the study was the simultaneous focus on both patient characteristics and ED process measures, and the subsequent development of a validated model by analyzing the predictors most associated with patients LWBS. The final model demonstrated “very good” discrimination with an AUC of 0.85, which suggests that the model can add significant value in “real time” in distinguishing between patients who LWBS vs patients who stay for treatment. We used a comparative cross-sectional design to study an ED population of large sample size linking patient factors and ED processes to the rate of LWBS. Moreover, we validated our model in a separate cohort after adjusting for seasonality. Several previous comparative studies have been performed focusing solely on patient characteristics associated with LWBS.^17^–^18^ The results of these studies corroborate our finding that younger age is associated with a higher number of patients LWBS. We also found significant associations with lower acuity (higher ESI level) and arrival as a “walk-in” rather than by EMS.

In terms of ED process measures, the number of boarders, patients in the waiting room and in treatment bays, arrival rate and “door-to-provider” times emerged as independent predictors in our study. Despite the fact that these measures appear to be closely correlated, we limited multicollinearity by studying a large sample of patients and using a regression model with backward selection. This methodology removed many of the process measures from the final model. Consequently, we believe that our final model represents stable and precise estimates of measures associated with LWBS. The significance is that real-time modification of any of the measures, independently of the rest, may be associated with a reduction in the number of patients LWBS. Moreover, identifying the key ED process measures from our model can lead to targeted hospital-wide strategies for improving day-to-day operations.

Hospital inefficiency and lack of patient flow result in an increase in the number of ED boarders, which emerged as a significant predictor of LWBS in our study. Optimized systems design and focused attention on the problem of boarding are required in the ED as well as on an institutional level in order to effect positive change.^19^ The “provider in triage” model was not implemented during the study period (nor has it been since completion of the study) since we believe the model is a resource intensive “work-around” of the true problem of ED boarding and poor hospital throughput. LWBS continues to be a challenge even in EDs that have implemented the provider in triage model albeit at a lower level. This model is far from ubiquitous and results in a net expense to the organization since the provider cannot bill for the service on the professional side (at least not in the state of Massachusetts). We continue to prefer to address the “real” problem rather than providing a less optimal and more expensive approach to emergency care.

While in-patient occupancy and LOS are important measures of patient flow in the hospital, we were not able to obtain these data in one-hour increments in our institution; we therefore could not include these measures in our model. Using an alternative method based on queueing theor*y* principles, Wiler et al also determined that reducing the number of patients boarding in the ED reduces the rate of LWBS.^10^ A regression analysis model focused exclusively on ED process measures determined that the total number of patients cared for in the ED, number of resuscitation and trauma patients, and the number of observation admissions explained only 52.8% of the variability in LWBS.^20^ ED occupancy (the number of registered patients divided by the number of licensed ED beds) of greater than 140% was shown to be an important contributor by other investigators.^21^ These results clearly speak to the importance of managing in-patient and ED flow and LOS as priorities when attempting to reduce the number of patients who LWBS.

We found that a “door-to-provider” time of greater than one hour appeared to be a point in time beyond which the LWBS rate significantly increased (p ≤ 0.003). While other investigators have found a range of delays of 30 minutes to two hours to be critical points in time, longer durations of the ED “front-end” process – from initial patient presentation to placement in an exam room – consistently predict that patients will LWBS at a higher rate. 12, 22–26 “Door-to-provider” times are increasingly important and have been greatly modified by administrative designs including fast-track care and providers in triage.^27^–^29^ Based on our results, we emphasize the importance of identifying critical “door-to-provider” times associated with LWBS, as this may guide current and future strategies. The acuity level of some patients who LWBS may actually have prompted admission had they decided to stay and complete a full evaluation. This is of particular concern for higher risk patients who occasionally experience adverse outcomes after LWBS from the ED.^30^ Accordingly, more of these patients re-present to the ED within 48 hours for care compared with patients who receive a complete evaluation and management at their initial ED presentation.^31^ Fortunately, patients with time-sensitive emergency conditions are typically assigned ESI levels that justifiably lead to early provider evaluation.^26^

The patient to RN ratios and the RN, attending physician, and emergency medicine resident staffing numbers reached statistical significance in univariable analysis but were not found to contribute significantly to our final model; moreover, these measures did not contribute to improvements in discriminating patients who LWBS vs those who were evaluated by a provider. Using a 24-hour rather than one-hour period as the unit of measure, investigators have previously found that, after controlling for ED volume, hospital occupancy and admission rate, fewer RN staffing hours are associated with a statistically significant increase in the number of patients who LWBS.^32^ Considerable variability in process measures occur in the ED over 24-hour periods, which is the reason we chose to collect data in one-hour increments.^33^

We emphasize that our results should not be interpreted to mean that patient to RN ratios and other measures of staffing are unimportant. Rather, they suggest that one or more other variables in the final model were more strongly correlated with the outcome and explained much of the association between the outcome and staffing measures. Moreover, physician and RN staffing may simply not demonstrate sufficient variability, compared with other measures, to be statistically significantly associated with the observed variability in the rate of LWBS; greater variability in a predictor will reduce the variability in the estimated beta coefficient.^34^ Ultimately, measures such as the number of boarders, patients in the waiting room, arrival rate and “door-to-provider” times, demonstrated stronger associations with patients LWBS in our study.

The ability to identify patients who are more likely to LWBS can highlight avenues for recovering potential lost revenue. Using this predictive model can help influence hospital administrators regarding the need to address boarding as a hospital-wide issue as opposed to an isolated ED problem. Moreover, the findings in this study can be used to advocate for additional staffing and creative workspace during hours when the arrival rates per hour are highest and when a surge in volume occurs. As mentioned earlier, this study highlights areas in which real-time modifications can result in significant changes in the rates of patients who LWBS. Strategies focused on reducing boarding, reducing the number of patients in the waiting room and treatment bays, arrival rate, and door-to-provider times have the opportunity to result in increased revenue and improved care and patient satisfaction.

## LIMITATIONS

Limitations apply to our study including that this was a single-center, cross-sectional study with separate derivation and validation cohorts that were collected from the same institution and time frame. We captured data for four seasonally adjusted months, rather than an entire 12-month period, but believe that our large sample sizes are representative of the overall annual experience. Moreover, we were able to obtain ED process measures at one-hour intervals, but not in smaller increments of time. It is theoretically possible, but not likely in our experience, that these measures vary significantly over smaller time periods. Important hospital-wide measures, such as in-patient occupancy, are calculated only once a day at midnight in our institution, thus rendering them relatively meaningless for our purpose.

We recognize that many of the variables assessed during model development are correlated with one another, which conceivably may induce multicollinearity and affect estimated standard errors of model coefficients. In severe cases, multicollinearity can produce very unstable and imprecise estimates of the standard errors, which may lead to unstable estimates of effect, wide CIs and misleading p-values. Multicollinearity, however, does not affect the utility of the regression model in estimating mean responses or making predictions.^35^ We applied remedies suggested by Vatcheva et al that focus on stabilizing the variance estimates.^36^ These include increasing the sample size, if possible, and removing one or more of the less important correlated variables. For the model development, our sample size was extremely large (n = 14,937) and we are therefore confident that our parameter estimates and standard errors are stable and precise. Secondly, our backward selection process removed many of the process and utilization variables, thus reducing the likelihood of severe multicollinearity. Third, we compared our final model with other possible models that may potentially capture LWBS risk. As such, we believe that our final model represents stable and precise estimates of factors associated with LWBS.

## CONCLUSION

The rate with which patients LWBS from the ED is frequently cited as a measure of operational efficiency. Based on our results, the numbers of patients in the waiting room and boarding inside the treatment area are positively associated with patients LWBS. Moreover, the arrival rate of new patients per hour is also associated with this outcome. We found that “door-to-provider” time plays an important role and can, at least in some measure, be reduced through administrative design. Not surprisingly, patients who LWBS tend to be younger in age, lower in acuity with a higher ESI score, and arrive ambulatory rather than by EMS.

## Figures and Tables

**Table:** 

-Asymptotic Normal-				
ROC	Obs	Area	Std. Err.	[95% Conf. Interval]
14,445	0.8542	0.0049	0.84324	0.86240

**Table 1:** Univariable analysis of patient characteristics – derivation model.

Patient Characteristics	Total (N = 14,937)	LWBS		P-value

		No (N = 13,815)	Yes (N = 1,122)	
Age, *mean (SD)*	49.5 (20.5)	50.4 (20.5)	38.1 (15.6)	< 0.001
Gender, n (%)				0.004
Female	7,976 (53.4)	7,330 (53.1)	646 (57.6)	
Male	6,961 (46.6)	6,485 (46.9)	476 (42.4)	
Acuity (ESI score), *mean (SD)*	2.65 (0.78)	2.60 (0.77)	3.18 (0.65)	<0.0001
Race/ethnicity, n (%)				< 0.001
White	7,893 (52.8)	7,507 (54.3)	386 (34.4)	
Hispanic	4,731 (31.7)	4,242 (30.7)	489 (43.6)	
Black	1,852 (12.4)	1,697 (12.3)	155 (13.8)	
Asian	178 (1.2)	162 (1.2)	16 (1.4)	
Other/unknown	283 (1.9)	207 (1.5)	76 (6.8)	
Arrival Mode, n (%)				< 0.001
Walk-in	8,198 (54.9)	7,234 (52.4)	964 (85.9)	
EMS	6,739 (45.1)	6,581 (47.6)	158 (14.1)	
Month, n (%)				< 0.001
September	3,647 (24.4)	3,396 (24.6)	251 (22.4)	
December	3,585 (24.0)	3,401 (24.6)	184 (16.4)	
March	4,020 (26.9)	3,558 (25.7)	462 (41.2)	
June	3,685 (24.7)	3,460 (25.1)	225 (20.1)	
6-hour time period, n (%)				<0.001
0001 – 6 am	1,858 (12.4)	1,754 (12.7)	104 (9.3)	
6 am – 12 pm	4,614 (30.9)	4,444 (32.2)	170 (15.2)	
12 pm – 6 pm	5,383 (36.0)	4,848 (35.1)	535 (47.7)	
6 pm – midnight	3,082 (20.6)	2,769 (20.0)	313 (27.9)	

*LWBS*, leaving without being seen; *SD*, standard deviation; *EMS*, emergency medical services; *ESI*, Emergency Severity Index.

**Table 2:** Clinical process variables – derivation model.

Process measures	Total (N = 14,937)	ED LWBS		P-value

		No (N = 13,815)	Yes (N = 1,122)	
Number, mean (SD)				
Waiting room	11.1 (7.7)	10.7 (7.6)	16.4 (7.5)	< 0.001
Treatment bays	83.8 (19.2)	83.2 (19.3)	91.9 (15.6)	< 0.001
Boarders	16.1 (7.8)	15.8 (7.7)	19.9 (7.8)	< 0.001
Attending physicians	4.7 (1.3)	4.7 (1.3)	5.0 (1.2)	< 0.001
Advanced practitioners	2.9 (0.7)	2.9 (0.7)	2.9 (0.7)	0.825
EM residents	4.0 (1.9)	4.0 (1.9)	4.2 (1.9)	< 0.001
Registered nurses	22.0 (3.2)	22.0 (3.2)	22.8 (2.7)	< 0.001
Patient care technicians	9.8 (2.3)	9.8 (2.3)	10.6 (2.0)	< 0.001
Patient - RN ratio, mean (SD)	4.2 (0.9)	4.2 (0.9)	4.7 (0.8)	< 0.001
Arrival rate/hour, mean (SD)	18.1 (6.7)	18.0 (6.7)	19.9 (6.4)	< 0.001
ED occupancy rate, mean (SD)	1.0 (0.2)	1.0 (0.2)	1.2 (0.2)	< 0.001
“Door-to-provider” time n (%)				<0.001
<30 mins	1912 (12.8)	1885 (13.6)	27 (2.4)	
30 mins – 59 mins	3628 (24.3)	3510 (25.4)	118 (10.5)	
60 mins – 89 mins	3527 (23.6)	3280 (23.7)	247 (22.0)	
90 mins – 119 mins	2674 (17.9)	2406 (17.4)	268 (23.9)	
120+ mins	3196 (21.4)	2734 (19.8)	462 (41.2)	

*ED*, emergency department; *LWBS*, leaving without being seen; *SD*, standard deviation; *EM*, emergency medicine; *RN*, registered nurse; *mins*, minutes.

**Table 3:** Final model: regression coefficients – derivation sample.

Explanatory variable	Odds ratio	95% CI	P-value
Age	0.98	[0.98 – 0.99]	< 0.001
Acuity	2.02	[1.85 – 2.21]	< 0.001
Arrival mode	0.29	[0.23 – 0.36]	< 0.001
Arrival rate/hour	1.03	[1.02– 1.05]	< 0.001
Hour (linear spline)			
0001 – 0600	1.0 (reference)		
0601 – 1200	0.28	[0.18 – 0.42]	< 0.001
1201 – 1800	0.4	[0.26 – 0.62]	<0.001
1801 – 0000	0.56	[0.38 – 0.81]	0.002
Race/ethnicity		-	
White (reference)	1.00		
Hispanic	1.24	[1.04– 1.48]	0.02
Black	1.19	[0.96– 1.49]	0.11
Asian	1.22	[0.63– 2.37]	0.55
Other/unknown	4.86	[3.42– 6.92]	< 0.001
Month			
Sep 2015	1.0 (reference)		
Dec 2015	0.78	[0.55 – 1.11]	0.18
Mar 2016	1.34	[0.98 – 1.84]	0.06
Jun 2016	1.02	[0.74 – 1.40]	0.90
No. in waiting room	1.05	[1.03– 1.06]	< 0.001
No. in treatment bays	1.01	[1.01 – 1.02]	< 0.001
No. of boarders	1.02	[1.01– 1.03]	0.001
Mean “door-to-provider” time			
<30 minutes	1.0 (reference)		
30 mins – 59 mins	1.34	[0.95 – 1.89]	0.09
60 mins – 89 mins	1.69	[1.20 – 2.39]	0.003
90 mins – 119 mins	1.87	[1.28 – 2.73]	0.001
120+ mins	1.99	[1.34 – 2.96]	0.001

a test of linear trend in odd ratios: p = 0.0002

*CI*, confidence interval, *mins*, minutes.

## References

[1] Pham JC, Ho GK, Hill PM (2009). National study of patient, visit, and hospital characteristics associated with leaving without being seen: predicting LWBS. Acad Emerg Med.

[2] Fry M, Thompson J, Chan A (2004). Patients regularly leave emergency departments before medical assessment: a study of did not wait patients, medical profile and outcome characteristics. Aust Emerg Nurs J.

[3] Baker DW, Steven CD, Brook RH (1991). Patient who leave a public hospital ED without being seen: causes and consequences. JAMA.

[4] Olanrewaju A, Soremekun MD, Biddinger PD (2012). Operational and financial impact of physician screening in the ED. Am J Emerg Med.

[5] Falvo T, Grove L, Stachura R (2007). The financial impact of ambulance diversions and patient elopements. Acad Emerg Med.

[6] Handel DA, Fu R, Vu E (2014). Association of emergency department and hospital characteristics with elopements and length of stay. J Emerg Med.

[7] Pitts SR, Vaughns FL, Gautreau MA (2014). A cross-sectional study of emergency department boarding practices in the United States. Acad Emerg Med.

[8] Hsiah RY, Asch SM, Weiss RE (2011). Hospital determinants of emergency department left without being seen rates. Ann Emerg Med.

[9] Stock LM, Bradley GE, Lewis RJ (1994). Patients who leave emergency departments without being seen by a physician: magnitude of the problem in Los Angeles County. Ann Emerg Med.

[10] Wiler JL, Bolandifar E, Griffey RT (2013). An emergency department flow model based on queueing theory principles. Acad Emerg Med.

[11] McCarthy ML, Ding R, Pines JM (2011). Comparison of methods for measuring crowding and its effects on length of stay in the emergency department. Acad Emerg Med.

[12] Pielsticker S, Whelan L, Arthur AO (2015). Identifying patient door-to-room goals to minimize left-without-being-seen rates. West J Emerg Med.

[13] Pan W (2001). Akaike’s information criterion in generalized estimating equations. Biometrics.

[14] Cui J (2007). QIC program and model selection in GEE analyses. Stata J.

[15] Austin PC, Steyerberg EW (2019). The integrated calibration index (ICI) and related metrics for quantifying the calibration of logistic regression models. Stat Med.

[16] Harrell FE (2015). Multivariate modeling strategies. Regression Modeling Strategies: With Applications to Linear Models, Logistic Regression and Survival Analysis.

[17] Sun BC, Binstadt ES, Pelletier A (2007). Characteristics and temporal trends of “left before being seen” visits in US emergency departments, 1995–2002. J Emerg Med.

[18] Ding R, McCarthy ML, Li G (2006). Patient who leave without being seen: their characteristics and history of emergency department use. Ann Emerg Med.

[19] Sharieff GQ, Burnell L, Cantonis M (2013). Improving emergency department time to provider, left-without-treatment rates and average length of stay. J Emerg Med.

[20] Hobbs D, Kunzman SC, Tandberg D (2000). Hospital factors associated with emergency department patients leaving without being seen. Am J Emerg Med.

[21] Polevoi SK, Quinn JV, Kramer NR (2005). Factors associated with patients who leave without being seen. Acad Emerg Med.

[22] Arendt KW, Sadosty AT, Weaver AL (2003). The left-without-being-seen patients: What would keep them from leaving?. Ann Emerg Med.

[23] McNamara KJ (1995). Patients leaving the emergency department without being seen by a physician: Is same-day follow-up indicated?. Am J Emerg Med.

[24] Spechbach H, Rochat J, Gaspoz JM (2019). Patient’s time perception in the waiting room of an ambulatory emergency unit: a cross-sectional study. BMC Emerg Med.

[25] Shaik SB, Jerrard DA, Witting MD (2012). How long are patients willing to wait before leaving without being seen?. West J Emerg Med.

[26] Jones P, LeFevre J, Harper A (2017). Effect of the shorter stays in emergency departments time target policy on key indicators of quality of care. NZ Med J.

[27] Yousefi M, Yousefi M, Fogliatto FS (2018). Simulating the behavior of patients who leave a public hospital emergency department without being seen by a physician: a cellular automaton and agent-based framework. Brazilian J Med and Biol Research.

[28] Rowe BH, Guo X, Villa-Roel C (2011). The role of triage liaison physicians on mitigating overcrowding in emergency departments: a systematic review. Acad Emerg Med.

[29] Holroyd BR, Bullard MJ, Latoszek K (2007). Impact of a triage liaison physician on emergency department overcrowding and throughput: a randomized controlled trial. Acad Emerg Med.

[30] Rowe BH, Channan P, Bullard M (2006). Characteristics of patients who leave emergency departments without being seen. Acad Emerg Med.

[31] Tropea J, Sundarajan V, Gorelik A (2012). Patient who leave without being seen in emergency departments: an analysis of predictive factors and outcomes. Acad Emerg Med.

[32] Ramsey Z, Palter JS, Hardwick J (2018). Decreased nursing staffing adversely affects emergency department throughput metrics. West J Emerg Med.

[33] Anderson D, Pimentel L, Golden B (2016). Drivers of ED efficiency: a statistical and cluster analysis of volume, staffing and operations. Am J Emerg Med.

[34] Vittinghoff E, Glidden DV, Shiboski SC (2012). Predictor selection. Regression Methods in Biostatistics: Linear, Logistic, Survival, and Repeated Measures Models.

[35] Kutner MH, Nachtsheim CJ, Neter J (2013). Multiple regression II Applied Linear Statistical Models.

[36] Vatcheva KP, Lee M, McCormick JB (2016). Multicollinearity in regression analyses conducted in epidemiologic studies. Epidemiol.

